# Development of chloroplast microsatellite markers for giant ragweed (*Ambrosia trifida*)

**DOI:** 10.1002/aps3.11313

**Published:** 2020-01-22

**Authors:** Himanshu Sharma, Jaakko Hyvönen, Péter Poczai

**Affiliations:** ^1^ Agri‐Biotechnology Divison National Agri‐Food Biotechnology Institute Mohali 140306 India; ^2^ Finnish Museum of Natural History (Botany) University of Helsinki P.O. Box 7 Helsinki FI‐00014 Finland; ^3^ Organismal Evolutionary Biology Research Program Faculty of Biology and Environmental Sciences Viikki Plant Science Centre University of Helsinki P.O. Box 65 FI‐00014 Finland

**Keywords:** *Ambrosia trifida*, Asteraceae, microsatellites, molecular markers, noxious weeds, plant invasions, plastid, polymorphism

## Abstract

**Premise:**

Plant invasions are increasing globally, and extensive study of the genetic background of the source and invading populations is needed to understand such biological processes. For this reason, chloroplast microsatellite markers were identified to explore the genetic diversity of the noxious weed *Ambrosia trifida* (Asteraceae).

**Methods and Results:**

The complete chloroplast genome of *A. trifida* was mined for microsatellite loci, and 15 novel chloroplast primers were identified to assess the genetic diversity of 49 *Ambrosia* samples. The number of alleles amplified ranged from two to six, with an average of 3.2 alleles per locus. Shannon's information index varied from 0.305 and 1.467, expected heterozygosity ranged from 0.178 to 0.645, and the polymorphism information content value ranged from 0.211 to 0.675 (average 0.428). The cross‐species transferability of the 15 microsatellite loci was also evaluated in four related *Ambrosia* species (*A. artemisiifolia*,* A. maritima*,* A. psilostachya*, and *A. tenuifolia*).

**Conclusions:**

The novel chloroplast microsatellite markers developed in the current study demonstrate substantial cross‐species transferability and will be helpful in future genetic diversity studies of *A. trifida* and related species.

Giant ragweed (*Ambrosia trifida* L.) belongs to the Asteraceae family (tribe Heliantheae, subtribe Ambrosiinae) together with 40–50 other ragweed (*Ambrosia* L.) species (Payne, 1964). The genus *Ambrosia* is native to North America with a center of diversity located in the Sonoran Desert (Martin et al., 2018). Giant ragweed is known as an invasive plant on the European continent together with common ragweed (*A. artemisiifolia* L.). Both species are noxious weeds and interfere with the growth development and establishment of various crops (Kong et al., 2007). The primary habitat of *A. trifida* is flood plains and ditch banks, but in the recent past it has spread to the Corn Belt in the United States, causing great economic losses. In addition to its competition with crops, *Ambrosia* pollen is responsible for allergic reactions in late summer and autumn (Ghosh et al., 1991) and constitutes a major health problem in North America and several countries in Europe. Urbanization and long‐distance transport capacity have enhanced the spread of ragweed species (Montagnani et al., 2017), while global heating and rising CO_2_ emissions are increasing the amount of *Ambrosia* pollen in the air (Wayne et al., 2002).

*Ambrosia* research is mostly concentrated on common ragweed, and therefore the population structure of other ragweed species is mostly unknown. For *A. artemisiifolia*, microsatellite markers were developed to determine the origins of invading populations in Europe (Gaudeul et al., 2011), and the history of the invasion was also investigated using herbarium specimens (Martin et al., 2014). Genomic resources are also under development to facilitate further research in ragweed genetics, e.g., the plastid genome of *A. trifida* (Sablok et al., 2019) and *A. artemisiifolia* (Amiryousefi et al., 2017; Nagy et al., 2017) have been sequenced. To the best of our knowledge, no reports have been published on chloroplast microsatellite markers for *A. trifida*, and the cross‐species transferability of microsatellite markers has not been investigated.

In the current study, we identified 15 novel chloroplast microsatellite markers, which will enrich the existing genomic resources for ragweeds, and we have successfully validated these markers in relevant giant ragweed populations and related species of *Ambrosia*.

## METHODS AND RESULTS

We searched the complete chloroplast genome of *A. trifida* (Sablok et al., 2019; GenBank accession number NC036810) for microsatellite loci. Simple sequence repeats (SSRs) were identified using MISA software (Thiel et al., 2003). Mononucleotide repeats were excluded from our search, and we applied a threshold based on minimum length criteria (unit size/minimum repeat time): six for di‐, four for tri‐, and three for tetra‐, penta‐, and hexanucleotide repeats, respectively. MISA allowed the identification and localization of perfect as well as compound microsatellites. Compound SSRs were considered repeats disrupted by a non‐repetitive length of 100‐bp sequence. Based on the flanking regions of the repeat regions, we designed primers for 15 cpSSR loci using the BatchPrimer3 version 1.0 web program (You et al., 2008). The parameters for designing PCR primers were as follows: (1) primer length ranging from 18 to 23 bp; (2) PCR product size range of 100–300 bp; (3) melting temperature between 50°C and 70°C, with 55°C as the optimum annealing temperature; and (4) a GC content of 40–70%, with an optimum of 50% (Table 1).

**Table 1:** The characteristics of 10 polymorphic and five monomorphic chloroplast microsatellite loci developed for *Ambrosia trifida*.

Locus^a^	Primer sequences (5′–3′)	Position	Repeat motif	Allele size range (bp)	Fluorescent label	GenBank accession no.
AART_MS2	F: GCAGTCAATTATGTGTCTCCT	*psb*C gene	(TTC)_3_	142–148	HEX	MN385584
	R: GTTCAAAATCACGATCAATTC					
AART_MS3	F: TGAAGTATGGAAGGAGATCAA	*rbc*L gene	(GGATAA)_3_	136–148	6‐FAM	MN385586
	R: TCGGCTCAATCCTTTTAGTA					
ATRI_MS9	F: GGAGTTCTTTCGTCTCAAAAT	*ycf*4‐*cem*A IGS	(ATT)_3_	138–144	TAMRA	MN385585
	R: TCCCTAATAGTCAAAAGCACA					
AART_MS5	F: CCTTGTACCATATTCCTAGCC	*pet*A‐*psb*J IGS	(TTCT)_4_	146–154	HEX	MN385587
	R: ACATTTCAATCTCACAATAGCA					
AART_MS9	F: CAGAATTCCAGTGTCTAATGG	*ndh*A intron	(TATC)_4_	140–160	6‐FAM	MN385588
	R: TGAGATTTTCATCTCATACGG					
ATRI_MS6	F: TACTGTTTAGGCAGAATACCG	*ycf*1 gene	(AGA)_3_	156–162	TAMRA	MN385593
	R: CCCATACATATAATCAACCGTA					
ATRI_MS8	F: GTAATGGCAATTGGAATGATA	*ndh*D gene	(TATT)_3_	145–161	HEX	MN385592
	R: AGGTAGAAAAATGGAGATGGA					
AART_MS11	F: CCAAAATTTGAGCGAAAATAG	*trn*L‐*rpl*32 IGS	(TTTA)_3_	159–167	6‐FAM	MN385591
	R: TTGGCAGTCCCTATTCTAATC					
AART_MP6	F: TCTTACAAGGAAAGGGAAA	*rpl*33‐*rps*18 IGS	(TATT)_4_	137–153	TAMRA	MN385589
	R: TAGTTTATTTCTTATCCCT					
AART_MP1	F: GCAGGTTAGATTCAAGCACTA	*trn*K‐*rps*16 IGS	(AATT)_3_	157–163	HEX	MN385590
	R: ATGCAGTGCCRATSYAACACA					
ATRI_MZ1	F: GGTAATCATTCAAATTAAGAACGGAAG	*trn*K intron	(AATG)_3_	200	—	—
	R: TTCTGAGAATATTTTATTCTTTCCGGC					
ATRI_MZ2	F: AGTGGACCTGACCCATTGAA	*trn*T‐*psb*D IGS	(TTTA)_3_	130	—	—
	R: CGACAAATTCTTGCGGAGTCC					
ATRI_MZ3	F: GATCTCGGGTGTGATGGTCA	*trn*S‐*psb*Z IGS	(ATCT)_3_	103	—	—
	R: CGGGGGAGAATGGAATTGGG					
ATRI_MZ4	F: TCGCTAATTGATCTTTACGGTGC	ycf3 intron II	(TTTC)_3_	163	—	—
	R: AGGGCTTTCTACATATGCATCGT					
ATRI_MZ5	F: TGGCGAAATTGGTAGACGCT	*trn*L intron	(AAAC)_3_	198	—	—
	R: GCTTCCATCGAGTCTCTGCA					

Note

IGS = intergenic spacer.

a The annealing temperature for all primers is 55°C except for AART_MP6 for which it is 52°C.

After primer design, amplification efficiency and polymorphism were evaluated using 29 *A. trifida* DNA samples from two different historical populations collected in Europe and North America. Cross‐amplification in the genus *Ambrosia* was assessed in five individuals each of *A. artemisiifolia*,* A. maritima* L., *A. psilostachya* DC., and *A. tenuifolia* Spreng. DNA samples were taken from the herbarium collection of the Finnish Museum of Natural History (Appendix 1). Leaf samples were rinsed with deionized water and 70% ethanol, and total genomic DNA was isolated using the E.Z.N.A. Plant Kit (Omega Bio‐tek, Norcross, Georgia, USA). Laboratory work was carried out in a separate DNA laboratory at the University of Helsinki. Blank samples were processed together with herbarium material during DNA extractions. Final concentrations were measured with a Qubit 2.0 Fluorometer (Thermo Fisher Scientific, Waltham, Massachusetts, USA). PCR amplification was carried out in a 20‐μL volume containing 20 ng of genomic DNA, 0.2 mM of dNTPs (Thermo Fisher Scientific), 2 μL 1× PCR buffer, 20 pM of each primer, and 0.25 units DyNAzyme DNA polymerase (Thermo Fisher Scientific). All reactions were performed in a MasterCycler ep96 (Eppendorf, Hamburg, Germany) with the following settings: 2 min of initial denaturation at 94°C; 35 cycles of denaturation for 30 s at 94°C, annealing for 1 min at 55°C, and extension for 2 min at 72°C; followed by a final extension for 5 min at 72°C. Amplification products were separated on 1.5% agarose gels (GE Healthcare, Chicago, Illinois, USA) using a 0.5× TBE buffer (220 V, 0.5 h) and stained with SYBR Safe DNA gel stain (Thermo Fisher Scientific) to validate successful amplifications.

After initial testing with three *A. trifida* and four *Ambrosia* species samples (*A. artemisiifolia*,* A. maritima*,* A. psilostachya*, and *A. tenuifolia*), the amplifications were repeated using fluorescently labeled primers applying the PCR protocol described above using all 49 *Ambrosia* samples. PCR products were analyzed on an ABI 3730XL DNA sequencer (Applied Biosystems, Foster City, California, USA) using a GeneScan 500 LIZ Size Standard (Applied Biosystems). The scoring of electropherograms was carried out with Geneious Prime (Biomatters Ltd., Auckland, New Zealand). All primers amplified successfully across the test individuals and 10 proved to be polymorphic, whereas five gave monomorphic patterns (Table 1). The polymorphic markers were used to evaluate the genetic diversity of 49 *Ambrosia* samples.

The number of alleles, effective number of alleles, and Shannon's information index were calculated with GenAlEx 6.5 (Peakall and Smouse, 2012), while expected heterozygosity and the polymorphism information content were calculated using iMEC (Amiryousefi et al., 2018). The number of alleles per locus ranged from two to six, the effective number of alleles ranged from 1.198 to 2.324, Shannon's information index ranged from 0.305 and 1.467, and the expected heterozygosity ranged from 0.178 to 0.645, while the polymorphism information content ranged from 0.211 to 0.675 (Table 2). The 10 polymorphic loci were also successfully amplified in 20 individuals (five individuals each) in the following four related species: *A. artemisiifolia*,* A. maritima*,* A. psilostachya*, and *A. tenuifolia* (Table 3, Appendix 1).

**Table 2:** Characteristics of 10 polymorphic chloroplast microsatellite markers in 29 individuals of giant ragweed (*Ambrosia trifida*).^a^

Locus	*Ambrosia trifida* (*N* = 29)				
	*A*	*A*_e_	*I*	*H*_e_	PIC
AART_MS2	3	1.624	0.675	0.413	0.478
AART_MS3	3	2.324	1.467	0.645	0.675
ATRI_MS9	3	2.112	0.808	0.573	0.597
AART_MS5	2	1.271	0.369	0.203	0.267
AART_MS9	6	1.654	0.911	0.426	0.501
ATRI_MS6	3	1.233	0.334	0.178	0.211
ATRI_MS8	4	1.198	0.305	0.446	0.523
AART_MS11	2	1.424	0.414	0.288	0.312
AART_MP6	4	1.806	0.897	0.446	0.489
AART_MP1	2	1.251	0.329	0.218	0.225
Mean	3.200	1.424	0.651	0.384	0.428

Note

*A* = number of alleles; *A*_e_ = effective number of alleles; *H*_e_ = expected heterozygosity; *I* = Shannon's information index; *N* = number of individuals sampled; PIC = polymorphism information content.

a Locality and voucher information are provided in Appendix 1.

**Table 3:** Results of cross‐amplification of 10 polymorphic chloroplast microsatellite markers developed for *Ambrosia trifida* in four related ragweed species.^a^ ^,^ ^b^

Locus	*A. artemisiifolia* (*N* = 5)	*A. psilostachya* (*N* = 5)	*A. tenuifolia* (*N* = 5)	*A. maritima* (*N* = 5)
AART_MS2	142–148	145–148	148–154	142–148
AART_MS3	142–148	148–154	142–154	142–148
ATRI_MS9	141–144	144–147	147–153	141–144
AART_MS5	146–154	150–158	154–162	150–154
AART_MS9	144–160	152–160	156–164	148–160
ATRI_MS6	159–162	156–162	159–168	159–162
ATRI_MS8	153–165	153–157	153–161	149–161
AART_MS11	159–171	163–175	163–175	159–171
AART_MP6	141–149	153–157	149–157	141–153
AART_MP1	161–163	163–171	159–167	157–163

Note

*N* = number of individuals sampled.

a Numbers shown represent the size in base pairs (bp) of the amplified fragments.

b Locality and voucher information are provided in Appendix 1.

## CONCLUSIONS

We used the recently sequenced plastid genome of *A. trifida* (Sablok et al., 2019) to develop and characterize 15 chloroplast microsatellite markers; these were then used to identify high genetic diversity among the analyzed giant ragweed samples. Overall population genetic variation was similar to that detected in common ragweed (*A. artemisiifolia*) in North America and in the Rhône‐Alpes region (Genton et al., 2005). Given the high level of polymorphism detected with the developed cpSSR primer set in the *A. trifida* population, the markers developed here should be suitable for further studies investigating the origin of invasive populations in Europe and studying the dynamics of invasion and modes of dispersal. Due to the high rate of cross‐amplification, the developed polymorphic cpSSR primers will likely be useful in intra‐ or interspecific genetic studies of the genus *Ambrosia*.

## Data Availability

All sequence information was deposited in the National Center for Biotechnology Information (NCBI) GenBank database (accession numbers MN385584–MN385593 and NC_036810.2; Table 1).

## Appendix 1 Sampling information for ragweed specimens used in this study.^a^

**Table:**

Species	Voucher no.	Collection year	Collection locality
*Ambrosia trifida* L.	H1651188	1936	Viipuri, Maaskola
	H1651189	1936	Viipuri, Maaskola
	H1645552	1938	Helsinki, botanical garden
	H1645551	ca. 1930–1935	Helsinki, botanical garden
	H1273891	1977	Helsinki, botanical garden
	H1725761	2001	Missouri, Washington State Park
	H1591544	1957	Arlington, Virginia, Anderson Hospital
	H1566112	1984	Cameron, Louisiana
	H1076406	1966	Middleton, Dane County, Wisconsin
	H1076407	1966	Middleton, Dane County, Wisconsin
	H1018948	1970	Towson, Baltimore County, Maryland
	H1139339	1975	Florence, Hampshire County, Massachusetts
	H1137652	1959	The Pas, Manitoba, Canada
	H1141347	1972	Lexington, Davidson County, North Carolina
	H1150975	1976	Saint‐Fulgence, Québec
	H1208063	1976	Saint‐Fulgence, Québec
	H1555799	1980	Saint Albans, Franklin County, Vermont
	H1645543	1939	Arnaud, Manitoba
	H1645544	1968	Saint‐Gédéon, Québec
	H1645548	1935	Rigaud, Québec
	H1645546	1961	Sioux Lookout, Ontario
	H1645545	1867	Middleton, Dane County, Wisconsin
	H1645547	1958	Saint‐Fulgence, Québec
	H1645550	1892	Wisconsin
	H1282191	1979	City of Thunder Bay, Ontario
	H1017127	1967	Mount Horeb, Dane County, Wisconsin
	H1589855	1988	Luhansk (then Voroshilovgrad), Ukraine
	H1581440	1972	Litomerice, Czech Republic
	H1645540	1902	Schleswig‐Holstein, Germany
*Ambrosia artemisiifolia* L.	H1070900	1968	Győr, Hungary
	H1216521	1976	Graz, Austria
	H1224407	1974	Solosnica, Slovakia
	H1679632	1992	Torino, Piemonte, Italy
	H1673036	1990	Anvers (Antwerp), Belgium
*Ambrosia maritima* L.	H1645521	1964	Massa, Tuscany, Italy
	H1645522	1964	Massa, Tuscany, Italy
	H1092472	1973	Berre‐l’Étang, France
	H1477643	1958	Punta Sabbioni, Venice, Italy
	H1475197	1981	Sanlúcar de Barrameda, Cadiz, Spain
*Ambrosia psilostachya* DC.	H1155299	1947	Noordwijk, the Netherlands
	H1247694	1979	Berlin, (West) Germany
	H1116344	1975	Anvers (Antwerp), Belgium
	H1594014	NA	NA
	H1467250	NA	NA
*Ambrosia tenuifolia* Spreng.	H1645534	1908	Chapelle St. Laurent Beaucaire, France
	H1487927	1967	Buenos Aires, Rio de Plata
	H1491053	1974	Buenos Aires, Florida, Calle General Roca
	H1486933	1967	Buenos Aires, Magdalena, Arroyo Juan Blanco
	H1486934	1967	Buenos Aires, Magdalena, Arroyo Juan Blanco

Note

NA = not available.

^a^ All vouchered specimens are deposited at the herbarium of the Finnish Museum of Natural History (H), University of Helsinki, Finland.
